# Associations between attitudes accepting of wife abuse and emotional abuse, forced heavy work, and food deprivation during pregnancy in Nepal: a cross-sectional study

**DOI:** 10.1080/16549716.2025.2603864

**Published:** 2026-01-02

**Authors:** Pratibha Manandhar, Pratibha Chalise, Poonam Rishal, Kunta Devi Pun, Jacquelyn Campbell, Lena Henriksen, Sunil Kumar Joshi, Mirjam Lukasse, Berit Schei, Jennifer Jean Infanti, Katarina Swahnberg

**Affiliations:** a Faculty of Medicine and Health Sciences, Department of Public Health and Nursing, Norwegian University of Science and Technology, Trondheim, Norway; b Department of Community Medicine, Kathmandu Medical College Public Limited (KMC), Kathmandu, Nepal; c Department of Nursing and Midwifery, Kathmandu University School of Medical Sciences (KUSMS), Dhulikhel, Nepal; d School of Nursing, Johns Hopkins University School of Nursing, Baltimore, MD, USA; e Faculty of Health Sciences, Department of Nursing and Health Promotion, Oslo Metropolitan University, Oslo, Norway; f Faculty of Health and Social Sciences, Center for Women’s, Family and Child Health, University of South-Eastern Norway, Kongsberg, Norway; g Department of Obstetrics and Gynecology, Women and Children’s Centre, St. Olavs University Hospital, Trondheim, Norway; h Faculty of Health and Life Sciences, Department of Health and Caring Sciences, Linnaeus University, Kalmar, Sweden

**Keywords:** family violence, gender norms, in-law perpetration, intimate partner violence, low- and middle-income country

## Abstract

**Background:**

Social norms and gendered power relationships contribute to the acceptability of ‘wife abuse’ – a common form of domestic violence globally.

**Objectives:**

To estimate the prevalence and overlap of emotional abuse, forced heavy work, and food deprivation during pregnancy and examine their association with women’s attitudes accepting of wife abuse in Nepal.

**Methods:**

Baseline data were used from a randomized controlled trial involving pregnant women aged 18 and older attending routine antenatal care at two public hospitals in Nepal between January 2023 and March 2025. Participants completed a color-coded audio computer assisted self-interview. Attitudes toward wife abuse were assessed using 16 items drawn from three existing instruments. Exploratory factor analysis identified three distinct attitudinal factors. The dependent variable was a composite indicator of emotional abuse, forced heavy work, or food deprivation (coded as present if any were reported). Associations between the three attitudinal factors and composite outcome were analyzed using multiple logistic regression, adjusting for relevant sociodemographic variables.

**Results:**

Emotional abuse, forced heavy work, or food deprivation was reported by 6.7% of participants. Acceptance of wife abuse for domestic shortcomings (Factor 1) was significantly associated with higher odds of experiencing abuse (aOR [1.75 (1.23–2.50)].

**Conclusions:**

Pregnant women who endorsed attitudes accepting wife abuse – particularly for perceived domestic shortcomings – had higher odds of experiencing emotional abuse, forced heavy work, or food deprivation. These findings highlight importance of addressing harmful gender norms within households and ensuring antenatal care settings include safe opportunities to identify and support women at risk.

## Paper Context

- **Main findings:** Pregnant women who viewed wife abuse as justifiable for perceived domestic shortcomings were more likely to report emotional abuse, forced heavy work, or food deprivation, while higher education was associated with lower risk.
- **Added knowledge:** The study shows that deeply rooted gender norms around household responsibilities shape women’s acceptance of abuse and their vulnerability during pregnancy.
- **Global health impact for policy and action:** The findings can guide community-based education (targeting stakeholders such as teachers, lawyers, religious leaders, health care providers, and women), antenatal care screening practices, and policy efforts aimed at transforming harmful gender norms and reducing domestic violence during pregnancy.

## Background

Violence against women is a violation of human rights related to gender discrimination and recognized as a significant social and health problem worldwide [1,2]. Domestic violence (DV), a common form of violence against women, is defined by Nepal’s Ministry of Law and Justice as ‘any physical, mental, sexual or economic harm perpetrated by one person on another with whom he or she has a family relationship, including acts of reprimand or emotional harm’ [3]. Despite growing awareness, DV remains pervasive and is still accepted as ‘normal’ in many societies [4]. Gender inequality, driven by patriarchal norms and unequal power dynamics between men and women, is a key driver of the incidence, recurrence, and severity of violence [5]. In many low- and middle-income countries (LMICs), widespread attitudes accepting of ‘wife abuse’ – a specific and prevalent form of DV – are reinforced by social norms and traditional gender roles [6]. When women perceive such violence as justifiable, this often reflects the internalization of patriarchal norms that shift responsibility onto women and normalize violence within households, rather than any genuine acceptance or endorsement of abuse. Importantly, these attitudes may also develop because of experiencing violence, indicating the possibility of reverse causality in the relationship between attitudes and abuse. Such normalization of violence as a routine part of women’s daily lives can further entrench tolerance for harmful practices and limit opportunities to challenge them [7–9].

Societies often assign roles and responsibilities to individuals and groups based on gender norms. In patriarchal contexts, men commonly retain authority over finances and decisions regarding mobility, while women are expected to assume primary responsibility for care-giving and domestic work. Within these roles, women may exercise autonomy and influence, but their authority is often constrained and undervalued in ways that reinforce male dominance [10]. This aligns with the conceptual framework developed by Rani et al., which suggests that transgressions of prescribed gender norms in patriarchal societies are major ‘triggers’ for DV [11].

Strong patriarchal norms are deeply embedded in Nepalese culture, as in many LMICs in Asia [11,12]. Women may feel ignored, trivialized, or undervalued, and may internalize these norms. Abuse is frequently viewed as normal, contributing to greater societal tolerance [13,14]. According to the Nepal Demographic Health Survey (NDHS) 2022, 19% of women agreed that a husband is justified in hitting or beating his wife under certain circumstances, including when she does not cook food to his taste, burns food, talks to other men, argues with him, doesn’t complete domestic chores, leaves the home without informing him, neglects children, or refuses sex [15].

Among the various forms of DV, emotional abuse is a major source of humiliation and may have even more severe health effects than physical abuse [16]. Although emotional abuse is common across many cultures, its recognition as violence can vary; and some behaviors, such as arguing or name-calling, may not be considered abusive unless they are repeated or cause profound harm [17]. In this study, emotional abuse was assessed using the culturally validated Nepalese Abuse Assessment Screen (N-AAS), which includes specific examples (e.g. humiliation, threats, restriction of contact with family) that women in Nepal themselves identified as harmful [18,19]. Pregnant women subjected to emotional abuse face increased risks of stress, anxiety, depression, and post traumatic stress disorder [20–22]. Threats by an intimate partner, one form of emotional abuse, have been significantly associated with low birth weight in newborns [23]. In Nepal, culturally-specific abuses, such as forced heavy work and food deprivation, have also been identified by our team through qualitative research and later validated clinically [18]. While such abuse may occur in other contexts, they carry risks during pregnancy, with significant implications for both maternal and fetal health.

According to the 2022 NDHS, 13% of women in Nepal reported experiencing emotional abuse by a spouse or other intimate partner [15]. A total of 12 systematic reviews and five meta-analyses have summarized the prevalence of intimate partner violence during pregnancy, showing wide variation in emotional abuse (1.8–67.4%) [24]. However, some of these reviews did not examine how emotional abuse overlaps with other forms of IPV, such as physical or sexual violence. In individual studies, the prevalence of emotional abuse during pregnancy was reported as 26% in Turkey, 24% in Pakistan, 26% in South Africa, and 31% in Tanzania [25–28]. Building on this evidence, the present study aims to find the prevalence of emotional abuse, forced heavy work, and food deprivation (EAFF) during pregnancy, explores their overlap, and examines their association with women’s attitudes accepting of wife abuse. It was hypothesized that stronger attitudes accepting of wife abuse, together with selected sociodemographic characteristics, would be associated with higher odds of experiencing EAFF during pregnancy.

## Methods

This cross-sectional study used baseline data from an ongoing randomized controlled trial (RCT) [29], collected between 29 January 2023 to 31 March 2025. All participants enrolled in the RCT during this period were included.

### Study sites

The study was carried out at two non-governmental institutions in Bagmati province, Nepal: Kathmandu Medical College (KMC), a teaching hospital located in central Kathmandu, and Dhulikhel Hospital (DH), a community-based tertiary hospital (28.9 km) east of Kathmandu. At KMC, all antenatal care (ANC) is provided by gynecologists and obstetricians, while at DH, midwives provide standard ANC for low-risk pregnancies, with obstetricians managing high-risk and complex cases.

### Inclusion criteria

All pregnant women aged 18 years or older, between 12 and 22 weeks of gestation, attending routine ANC at either DH or KMC were invited to participate.

### Exclusion criteria

Women who did not provide consent, younger than 18 years, and pregnant women experiencing significant health issues, those not able to comprehend Nepali language and those who chose not to participate were excluded.

### Data collection methods

Upon obtaining verbal and electronic written consent, participants self-completed a study questionnaire using an electronic data collection format known as colour-coded audio computer-assisted self-interview (C-ACASI) [30]. More detailed information on participant recruitment is available in the Addressing Domestic Violence in Antenatal Care Environments 2 (ADVANCE 2) study protocol [29]. An encrypted cloud server that was self-hosted housed all the data that was gathered. To ensure privacy and prevent influence from family members, all participants completed the C-ACASI questionnaire alone in a private room using a tablet and headphones.

### Dependent variables

#### Emotional abuse, forced heavy work, and food deprivation

This study focuses on three forms of DV: EAFF (emotional abuse, forced heavy work, and food deprivation). These were measured using the validated Nepalese version of the Abuse Assessment Screening instrument (N-AAS) [18] (see Table 1).

**Table 1.** Questionnaire measures of domestic violence used in the study.

Types of abuse	Questions inquired	Response	Perpetrators (multiple responses)
Emotional abuse	‘*Since you’ve been married or living with a partner, have you been emotionally abused by someone in the family?’ (Examples: Emotional abuse includes such acts as saying or doing something to humiliate you intentionally in front of others, the use of abusive words directed at you, threatening to hurt or harm you and your child/children, insulting you, deliberately making you feel bad about yourself, prohibiting you from visiting your mother’s home, etc.)*	Yes/No	husband, ex-husband, partner, mother-in-law, father-in-law, and other family members
Forced heavy work	‘*Since you’ve been pregnant, have you been forced by someone in the family to do heavy physical work despite feeling unwell or exhausted?’ (Examples: Forced heavy physical work includes activities such as being made to carry a 20 liter of water jar or load of hay, forced to sit for a long period of time while washing clothes, lift sacks of rice, prolonged walking, etc.).*	Yes/No	husband, ex-husband, partner, mother-in-law, father-in-law, and other family members
Food deprivation	*‘Since you’ve been pregnant, has someone in the family given you insufficient or no food to eat despite the availability of food and money in the household?’ (Examples: not providing nutritious food such as eggs, fish, meat, fruits or hiding those foods).*	Yes/No	husband, ex-husband, partner, mother-in-law, father-in-law, and other family members

A composite variable was coded as a present if participants reported experiencing any one or more of the three forms of abuse. In addition, a mutually exclusive variable with seven categories was created to capture all possible combinations of EAFF, allowing for analysis of overlap and co-occurrence (refer to Table 2).

**Table 2.** Socio-demographic characteristics of the participants (*n* = 2909).

Socio-demographic characteristics		Total n (%)
Study site	Dhulikhel Hospital	1421 (48.8)
	Kathmandu Medical College	1488 (51.2)
Age (years)	18–24	719 (24.7)
	25–29	1181 (40.6)
	30–49	1009 (34.7)
Primigravid	Yes	1328 (45.7)
	No	1581 (54.3)
Currently married	Yes	2892 (99.4)
	No	17 (0.6)
Independent income	Yes	1206 (41.5)
	No	1703 (58.5)
Education	Low education	619 (21.3)
	High education	2290 (78.7)
Family type	Nuclear	1648 (56.7)
	Joint	1261 (43.3)
Ethnic group	Adhibasi/Janajati	1034 (35.5)
	Brahmin/Chhetri	1519 (52.2)
	Dalit	130 (4.5)
	Madhesi/Muslim	102 (3.5)
	Others	124 (4.3)
Settlement	Rural	813 (27.9)
	Urban	2096 (72.1)

### Independent variables

#### Sociodemographic characteristics

Sociodemographic variables included age and ethnicity, categorized as Brahmin/Chhetri/Thakuri, Adhibasi/Janajati, Dalit, and others (Madhesi/Muslims). Settlement was categorized as urban or rural residence. Education was based on actual years of schooling and re-categorized as low education (completed schooling up to grade 5) or high education (completed class 6 and higher) for the binary variable used in regression for statistical analysis. Gravida was classified as primigravida (first pregnancy) or multigravida (more than one pregnancy). Independent income was assessed by asking participants if they had their own income (Yes/No). Family structure had two categories: nuclear (husband and wife and unmarried children) or joint (husband/wife, unmarried/married adult children, and/or in-laws). Marital status was recorded as currently married (Yes/No).

#### Attitudes on acceptance of wife abuse

The study used 16 questions to assess attitudes toward the acceptance of wife abuse. The questions were selected from three instruments: the Social Acceptance of Wife Abuse (SAWAS), validated in Bangladesh [31], the World Health Organization questionnaire on attitudes accepting of wife abuse [4], and the Nepal Demographic Health Survey 2022 [15].

Pregnant women were asked whether a husband is justified in hitting or beating his wife under specific conditions, such as:

she fails to prepare meals in time, she burns the meals, she fails to prepare tasty meals, she chats with a man, she argues with her husband, she does not complete her household work to his satisfaction, she disobeys him, she refuses to have sex/perform any sexual acts with him, she asks him whether he has other girlfriends, he suspects that she is unfaithful, he finds out that she has been unfaithful, she brings less or no dowry, she gives birth to daughters only and no son, his family tells him to do it, she neglects the children, she goes out without telling him.

The attitudinal items explicitly reference husband-to-wife violence (i.e. whether a husband is justified in hitting or beating his wife under specified circumstances). In contrast, the abuse indicators used as outcome variables capture perpetration by any family member, including husbands, mothers-in-law, fathers-in-law, and other relatives. The husband-referent attitudinal items were retained because they are widely used in DHS/WHO surveys and are theorized to represent broader normative acceptance of violence within patrilocal and extended-family households, where multiple actors may exert control over pregnant women.

### Exploratory factor analysis (EFA)

EFA was performed to identify the underlying factor structure of the ‘*acceptance of wife abuse’* items. EFA was selected because it is appropriate for examining latent constructions when the factor structure is unknown or when adapting scales to a new cultural or contextual context.

Factors were extracted using Varimax rotation, an orthogonal technique that maximizes the variance of squared loadings across variables simplifying interpretation by assuming that the extracted factors are independent. Following established methodological recommendations, factor loadings of 0.50 were considered meaningful for interpretation [32].

#### Factor structure

A three-factor structure was clearly visible in the Varimax-rotated solution. Items with factor loadings ranging from 0.56 to 0.71 were included in Factor 1, indicating a coherent group of variables measuring a shared underlying dimension. A second distinct dimension was reflected in Factor 2, which included items with loadings between 0.68 and 0.76. A third construction, Factor 3, was made up of items with loadings ranging from 0.61 to 0.76 (refer to Supplementary Table S1).

Factor 1 included nine items (*she fails to prepare meals in time, she burns the meals, she fails to prepare tasty meals, she chats with a man, she argues with her husband, she does not complete her household work to his satisfaction, she disobeys him, she refuses to have sex/perform any sexual acts with him, she asks him whether he has other girlfriends*) and was labeled ‘Domestic shortcomings’.

Factor 2 consisted of three items (*she brings little or no dowry, she gives birth to daughters only and no son, his family telling him to do it*) and was labelled ‘Marital and patriarchal expectations’.

Factor 3 included four questions (*he suspects that she is unfaithful, he finds out that she has been unfaithful, she neglects the children, she goes out without telling him*) and was labelled ‘Transgressing expected female family roles’.

### Analytical strategies

Frequencies and percentages were calculated to describe the participants’ sociodemographic characteristics, the individual prevalence of emotional abuse, forced heavy work, and food deprivation, their combined prevalence (any positive response to EAFF), and the prevalence of attitudinal acceptance of wife abuse. For the three factors capturing different dimensions of acceptance of wife abuse, responses were re-coded into binary variables: 1 for indicating acceptance and 0 for non-acceptance. Responses of ‘I don’t know’ were merged with ‘Yes,’ following prior literature indicating that uncertainty or hesitation is often indicative of implicit acceptance in similar settings.

Binary logistic regression was first performed to estimate crude odds ratios (COR) and adjusted odds ratios (aOR), with 95% confidence intervals (CI) for the association between EAFF and participants’ sociodemographic variables as well as the three attitudinal factors (domestic shortcomings [factor 1], marital and patriarchal expectations [factor 2], and transgressions of expected female family roles [factor 3]).

All sociodemographic variables were initially examined in bivariable analyses. Variables showing statistically significant associations with EAFF were retained for the multivariable model. Religion and wealth index were not collected in the parent trial, and variables with very limited variability or no bivariable association (e.g. marital status, independent income) were excluded to avoid overfitting given the low outcome prevalence (6.7%). The final adjusted model therefore included age, education, family type, settlement, and the three attitudinal factors.

Subsequently, multivariable logistic regression was conducted to assess the adjusted odds ratios (aOR) and 95% CI between age, education and Factors of attitude towards acceptance of wife abuse (selected a priori based previous literature) [11,31]. A p-value of <0.05 was considered statistically significant. The Kaiser-Meyer-Olkin (KMO) measure of sampling adequacy and Bartlett’s test of sphericity were used to evaluate the suitability of the data for factor analysis. The results indicated that the data were appropriate (KMO = 0.924; Bartlett’s test χ^2^ (120) 16,698.63 *p* < 0.001). The Cronbach’s Alpha for Factor 1 = .861, Factor 2 = .763, Factor 3 = .705, with no item below 0.50. Factor scores were generated using the regression method in SPSS and entered as predictors in the binary logistic regression.

Statistical analyses were conducted using SPSS Version 30.0.0.0.

### Ethical considerations

Prior to collecting data, formal ethical approvals were obtained from Nepal Health Research Council, Nepal (Ref: 2395) and The Regional Committee for Medical and Health Research Ethics of Central Norway (REK) (Ref: 178,092). The Declaration of Helsinki’s guiding principles were followed in the conduct of this study [33]. The study comprised a two-step consent process. First, verbal agreement for participation was obtained, which involved explaining the study objective to the participants and asking if they were willing to receive further and more detailed information. The second step was obtaining electronic consent in the C-ACASI.

## Results

Table 1 presents the questionnaire items used to measure emotional abuse, forced heavy work, and food deprivation in the study.

Table 2 provides the sociodemographic traits of the participants. Nearly half (48.8%) were recruited from Dhulikhel Hospital (DH) and 51.2% from Kathmandu Medical College (KMC). The largest age group, with 40.6%, was 25–29 years old. The Brahmin/Chhetri ethnic group accounted for most participants (52%), and a sizable portion (78%) had higher education.

Table 3 presents the prevalence of emotional abuse, forced heavy work, and food deprivation. Overall, 6.7% of participants reported at least one of these forms of abuse, whereas emotional abuse alone was reported by 4.0%.

**Table 3.** Prevalence of individual and overlapping forms of abuse among pregnant women (*n* = 2909).

Types of abuse reported	Yes n (%)	No n (%)
Emotional abuse only	116 (4.0)	2793 (96.0)
Forced heavy work only	24 (0.8)	2885 (99.2)
Food deprivation only	22 (0.8)	2887 (99.2)
Emotional abuse + Forced heavy work	18 (0.6)	2891 (99.4)
Emotional abuse + Food deprivation	5 (0.2)	2904 (99.4)
Forced heavy work + Food deprivation	0 (0)	2909 (100)
Emotional abuse + Forced heavy work + Food deprivation	8 (0.3)	2901 (99.7)
Any of the above (≥1 type of abuse reported)	193 (6.7)	2716 (93.3)

Note: ‘Any of the above’ refers to women reporting at least one of the three forms of abuse (EAFF), either alone or in combination.

Table 4 shows the types of perpetrators of EAFF among pregnant women. Mothers-in-law and other family members were the most frequently reported perpetrators.

**Table 4.** Perpetrators of emotional abuse, forced heavy work and food deprivation reported by pregnant women.

Type of abuse	Perpetrator	N (%)
Emotional abuse	Husband	37 (25.2)
	Ex-husband	4 (2.7)
	Partner	1 (0.7)
	Mother-in-law	55 (37.4)
	Father-in-law	24 (16.3)
	Other family members	74 (50.3)
**Total**		**195 (100)**
Forced heavy work	Husband	14 (28)
	Ex-husband	1 (2)
	Partner	1 (2)
	Mother-in-law	19 (38)
	Father-in-law	6 (12)
	Other family members	23 (46.0)
**Total**		**64 (100)**
Food deprivation	Husband	7 (20)
	Ex-husband	0
	Partner	1 (2.9)
	Mother-in-law	13 (37.1)
	Father-in-law	1 (2.9)
	Other family members	17 (48.5)
**Total**		**39 (100)**

Note: Multiple responses permitted. The total number of perpetrators exceeds the number of women affected because some women reported emotional abuse, forced heavy work, or food deprivation by more than one family member.

Table 5 shows the prevalence of individual acceptance of wife abuse, along with their grouping into three attitudinal factors. One individual item, ‘He finds out that she has been unfaithful,’ and Factor 3: ‘Transgressing expected female family roles’ had the highest prevalence − 48.3% and 61.9% respectively, compared to other individual as well as group factors. A significant association was seen between EAFF and nine of the 16 items. Several individual items measuring acceptance of wife abuse were statistically significant (*p* < 0.05) with EAFF prior to regression analysis.

**Table 5.** Prevalence of acceptance of wife abuse overall and by attitudinal factors: domestic shortcomings, marital and patriarchal expectations, and transgressing expected female family roles (*n* = 2909).

Factor/Item	Yes n (%)	No n (%)
**Factor 1: Domestic shortcomings**		
She fails to prepare meals in time	182 (6.3)	2727 (93.7)
She burns the meals	114 (3.9)	2795 (96.1)
She fails to prepare tasty meals	164 (5.6)	2745 (94.4)
She chats with a man	365 (12.5)	2544 (87.5)
She argues with her husband	220 (7.6)	2689 (92.4)
She does not complete her household work to his satisfaction	224 (7.7)	2685 (92.3)
She disobeys him	315 (10.8)	2594 (89.2)
She refuses to have sex/perform any sexual acts with him	166 (5.7)	2743 (94.3)
She asks him whether he has other girlfriends	272 (9.4)	2637 (90.6)
**Factor 2: Marital and patriarchal expectations**		
She brings less or no dowry	99 (3.4)	2810 (96.5)
She gives birth to daughters only and no son	103 (3.5)	2806 (96.5)
His family tells him to do it	136 (4.7)	2773 (95.3)
**Factor 3: Transgressing expected female family roles**		
He suspects that she is unfaithful	850 (29.2)	2059 (70.8)
He finds out that she has been unfaithful	1404 (48.3)	1505 (51.7)
She neglects the children	1110 (38.2)	1799 (61.8)
She goes out without telling him	685 (23.5)	2224 (76.5)

Table 6 presents results from a binary logistic regression analysis examining associations between EAFF, sociodemographic characteristics (controlled), and attitudes accepting of wife abuse. Pregnant women who accepted domestic shortcomings (factor 1) as justifications for wife abuse were 1.75 times more likely to report experiencing emotional abuse, forced heavy work, and food deprivation [aOR [1.75 (1.23–2.50)]. Completed higher education was protective against emotional abuse or forced heavy work or food deprivation [aOR [0.44 (0.31–0.61)], not shown in Table 6.

**Table 6.** Association between attitudes accepting of wife abuse (factor 1, factor 2, factor 3), sociodemographic variables, and emotional abuse or forced heavy work or food deprivation (*n* = 2909).

Independent variable	Sig.	COR (95% CI)	Sig.	AOR (95% CI)
Factor 1 (Domestic shortcomings)	**<.001**	**1.98 (1.46–2.67)**	.**002**	**1.75 (1.23–2.50)**
Factor 2 (Marital and patriarchal expectations)	.21	1.38 (.83–2.29)	.50	.82 (.47–1.43)
Factor 3 (Transgressing expected female family roles)	.59	1.08 (.80–1.47)	.20	.80 (.57–1.12)

Note: The final model presents attitudinal predictors only and is adjusted for age, marital status, education, family type, and settlement. The significant value is provided in bold font.

## Discussion

This study examined the association between EAFF and attitudes accepting of wife abuse among pregnant women attending antenatal care in two hospitals in Nepal. While 4.0% of participants reported experiencing emotional abuse, fewer than 2% reported forced heavy work or food deprivation during their pregnancy. Most women who experienced emotional abuse reported it alone, whereas only a smaller proportion reported emotional abuse together with food deprivation or forced heavy work, and none reported food deprivation and forced heavy work without emotional abuse. These findings highlight forms of abuse that may be substantially under-recognized or under-reported, particularly in low-resource settings where such practices may be normalized by poverty or gendered expectations [19]. Forced heavy work is often seen as part of women’s expected domestic duties, and food deprivation may be misinterpreted as scarcity rather than intentional harm. As shown in our earlier qualitative research [19], many women consider these practices ordinary or inevitable. During pregnancy, however, when rest and increased nutrition are essential, such norms may heighten maternal and fetal risks.

One explanation for the low reporting of forced heavy work and food deprivation may be the lack of routine inquiry into these abuses, despite their clear relevance to maternal health. These forms of abuse were identified in qualitative research conducted in rural Nepal [19], whereas participants in the present study were primarily from urban and peri-urban nuclear-family settings, where women may have somewhat greater autonomy. In extended-family households, decisions regarding food, workload, and healthcare are often made by husband or in-laws, making it more difficult for women to recognize or report harmful practices – especially when perpetrated by female relatives. For example, mothers-in-law often hold significant authority over household resources and may pressure or even coerce pregnant women into performing heavy labor or deny them adequate food [19]. It is also possible that the lower prevalence reflect true contextual differences. Many participants lived in urban or peri-urban nuclear households, where husbands often migrate for work and women may have greater autonomy. These shifts can limit opportunities for in-laws or other relatives to impose heavy labor or restrict food during pregnancy. One explanation for the low reporting could be the lack of routine inquiry into forced heavy work and food deprivation.” It could also be true prevalence.

Studies in Malaysia, Pakistan, and South Africa, and other LMICS report much higher prevalence of emotional abuse during pregnancy (26%–43%) [27,34,35]. In patriarchal contexts, DV is frequently normalized and understood as a legitimate form of discipline, not only by husbands but also by other family members. This reflects broader social structures in which power extend beyond the marital relationship [31]. The relatively low prevalence reported in this study may reflect true differences in context, under reporting, or both. Emotional abuse may be difficult for women to identify as ‘abuse,’ particularly if it reflects longstanding household dynamics, or they may choose not to disclose it due to shame, fear, or the heightened vulnerability or dependence on their partners associated with pregnancy. Distinguishing underreporting from true lower prevalence remains challenging, but both possibilities warrant consideration.

Consistent with Nepal’s patrilocal traditions, in which women move into their husband’s household after marriage, this study found that mothers-in-law and other family members were the most common perpetrators of EAFF. This aligns with research from India and other South Asian contexts showing that in-laws frequently perpetrate verbal abuse, harassment, forced labor, and food deprivation during the perinatal period [36–39]. Although the attitudinal items in this study focused specifically on husband-to-wife violence, abuse perpetrated by in-laws was common. This underscores how patriarchal norms are embedded within extended households and shape women’s experiences of abuse more broadly. These findings also suggest that interventions – particularly within ANC and community settings – may need to engage influential household members such as mothers-in-law, who often control pregnant women’s workload, food access, and mobility.

Attitudes accepting of wife abuse for perceived domestic shortcomings (Factor 1) were significantly associated with higher odds of reporting EAFF. Several interconnected cultural and psychological mechanisms may explain this association. In highly patriarchal contexts, norms that reinforce men’s authority and women’s subservience contribute to women’s acceptance of violence and their reduced likelihood of challenging abuse [40–42]. Although the attitudinal questions referenced husband-perpetrated violence, these attitudes may reflect broader normative acceptance of hierarchical family structures in which multiple relatives – including in-laws – exercise control. This may help explain the association even when the perpetrator was not the husband.

Overall, 6.7% of pregnant women reported at least one form of EAFF. These results suggest that some women experience multiple forms of abuse concurrently, a pattern not always captured in previous studies. Attitudes accepting of wife abuse may contribute to the normalization and internalization of such violence, making it more difficult for women to recognize abusive behaviors or seek support [43].

Higher education was associated with significantly lower odds of reporting EAFF [aOR 0.44 (0.3–0.61)], consistent with previous research from Nepal and other LMIC settings [10,11,40,44,45]. Education may reduce women’s vulnerability through increased awareness of rights, improved autonomy, and reduced financial dependence.

These findings suggest that ANC visits could serve as a practical point of contact for identifying and supporting women experiencing these forms of abuse. In Nepal, pregnancy is often one of the few times women can access health services independently. Offering a brief, confidential screening opportunity during ANC – such as giving women a private space to complete a short safety questionnaire – may help facilitate disclosure and connect women to appropriate support.

Given that many perpetrators in this study were in-laws or other family members, community-based platforms that already engage husbands and household decision-makers – such as ward-level health promotion activities or mothers’ group meetings – may also offer opportunities to address harmful norms within families.

### Study strengths and limitations

The study benefited from the use of validated measures for violence and attitudes accepting of wife abuse, ensuring contextual and cultural appropriateness. The factor-analytic approach supported the reliability of the attitudinal scale in the local context. The large and diverse sample drawn from two tertiary hospitals and spanning multiple ethnic and sociodemographic groups enhances generalizability within similar urban and peri-urban populations in Nepal.

Some limitations should be considered. First, under-reporting is likely, given the sensitivity of the subject matter and challenges in recognizing emotional abuse, forced heavy work, or food deprivation as forms of violence. Second, combining attitudinal items from three instruments may have introduced measurement inconsistencies. Additionally, ‘don’t know’ responses are difficult to interpret, as they may reflect fear, uncertainty, or passive acceptance. We grouped ‘don’t know’ with ‘yes’ based on prior literature suggesting that hesitation may reflect implicit acceptance [45], although alternative interpretations are possible.

Another limitation is that attitudes were measured specifically in relation to a husband’s behavior, whereas reports of abuse included husbands, in-laws, and other relatives. This mismatch may weaken observed associations and should be considered when interpreting results. Future research could incorporate attitudinal items referencing other family members, or analyze subgroups by perpetrator when sample sizes permit.

Finally, the cross-sectional design prevents establishing causality or temporal ordering between attitudes and experiences of abuse. All data were self-reported and may be influenced by social desirability or recall bias. Findings may not generalize to non-pregnant women, although the insights remain relevant for many LMIC settings with comparable gender norms and social structures.

## Conclusions

This study found that pregnant women in Nepal experience emotional abuse, forced heavy work, or food deprivation, and that these abuses are associated with attitudes accepting of wife abuse for perceived domestic shortcomings, as well as with lower levels of education. These findings underscore the importance of addressing harmful gender norms that contribute to the acceptance and normalization of such behaviors. Strengthening safe and confidential opportunities for inquiry during routine ANC may help identify women at risk and support appropriate referral. Broader community-based efforts to challenge patriarchal norms and promote women’s rights may further contribute to preventing these forms of abuse.

## Supplementary Material

STROBE_Checklist.docx

Supplementary_file_containing_all_the_supplememtary_figures_and_tables.docx

Tables_with_their_respective_legends_clean copy.docx

## Data Availability

The datasets generated and analyzed during the current study are not publicly available due to their inclusion in baseline assessments of a larger and ongoing randomized controlled trial, where the data remains blinded and incomplete in its final form. Additionally, the dataset contains sensitive information that has not yet been fully de-identified for sharing. However, a de-identified version can be made available from the corresponding author upon reasonable request.
