# Correction

**DOI:** 10.1080/16549716.2026.2629743

**Published:** 2026-02-26

**Article title**: Associations between attitudes accepting of wife abuse and emotional abuse, forced heavy work, and food deprivation during pregnancy in Nepal: a cross-sectional study

**Authors**: Pratibha Manandhar, Pratibha Chalise, Poonam Rishal, Kunta Devi Pun, Jacquelyn Campbell, Lena Henriksen, Sunil Kumar Joshi, Mirjam Lukasse, Berit Schei, Jennifer Jean Infanti, Katarina Swahnberg, and on behalf of the ADVANCE 2 study team

**Journal**: *Global Health Action*

**Bibliometrics**: Volume 19, Number 1, e2603864

**DOI**: http://dx.doi.org/10.1080/16549716.2025.2603864

In the published article, two minor reference-related corrections are noted. The authors regret these errors.

1. First, reference [4] cited the *WHO multi-country study on women’s health and domestic violence against women* as a report. This reference has been replaced with the corresponding peer-reviewed journal publication:

García-Moreno C, Jansen HAFM, Ellsberg M, Heise L, Watts C. *Prevalence of intimate partner violence: findings from the WHO multi-country study on women’s health and domestic violence*. The Lancet. 2006;368(9543):1260–1269.

2. Second, the article incorrectly referred to the Nepal Demographic and Health Survey (NDHS) 2022 questionnaire reference [15]. The correct reference is NDHS 2016. This article only used the questionnaire of NDHS 2016. No NDHS dataset was used in the study, and this correction does not affect the methods, analyses, results, or conclusions of the article. The reference has been replaced with:

Ministry of Health and Population (MOHP). New ERA, and ICF International Inc. Nepal Demographic and Health Survey 2016. [Internet]. Ministry of Health and Population, New ERA, and ICF International; 2017 [cited on 2023 April 28]. Available from: https://dhsprogram.com/publications/publication-fr336-dhs-final-reports.cfm.

