# Supplementary material for: Associations between attitudes accepting of wife abuse and emotional abuse, forced heavy work, and food deprivation during pregnancy in Nepal: a cross-sectional study
Source: Glob Health Action. 2026 Jan 2;19(1):2603864. doi: 10.1080/16549716.2025.2603864 (PMC12777793; doi:10.1080/16549716.2025.2603864)
Supplement: STROBE_Checklist.docx [file ZGHA_A_2603864_SM5399.docx]

STROBE Checklist Template

# Title and Abstract

| Checklist Item | Page Number(s) |
| --- | --- |
| (a) Indicate the study’s design with a commonly used term in the title or abstract | 3 |
| (b) Provide an informative and balanced summary in the abstract | 3 |

# Introduction

| Checklist Item | Page Number(s) |
| --- | --- |
| Background/rationale: Explain scientific background and rationale | 4-6 |
| Objectives: State specific objectives, including hypotheses | 6 |

# Methods

| Checklist Item | Page Number(s) |
| --- | --- |
| Study design: Present key elements of study design early | 6 |
| Setting: Describe setting, locations, and dates, including recruitment/follow-up | 6 |
| Participants: (a) Give eligibility criteria, sources, methods of selection | 7-9 |
| Participants: (b) For matched studies, give matching criteria (if applicable) | NA |
| Variables: Clearly define outcomes, exposures, predictors, confounders, effect modifiers | 8-10 |
| Data sources/measurement: For each variable, give data sources and methods of assessment | 7, 8, 9, 10,11 |
| Bias: Describe efforts to address potential sources of bias | 20 |
| Study size: Explain how study size was determined | 6 |
| Quantitative variables: Explain handling of quantitative variables | 7-11 |
| Statistical methods: (a) Describe all statistical methods, including control for confounding | 11 |
| Statistical methods: (b) Describe methods for subgroups and interactions | 11 |
| Statistical methods: (c) Explain how missing data were addressed | NA |
| Statistical methods: (d) Specific study type methods (cohort/case-control/cross-sectional) | Cross sectional method |
| Statistical methods: (e) Sensitivity analyses (if applicable) | Not applicable |

# Results

| Checklist Item | Page Number(s) |
| --- | --- |
| Participants: Report numbers at each stage (eligibility, inclusion, follow-up, analysis) | 12-16 |
| Descriptive data: Characteristics of study participants, exposures, missing data | 13 |
| Outcome data: Report numbers of outcome events/summary measures | 13-16 |
| Main results: (a) Unadjusted and adjusted estimates, confounders accounted for | 15-16 |
| Main results: (b) Report category boundaries when continuous variables are categorized | All variables are in categorical variable (page no. 7-10) |
| Main results: (c) Translate estimates into absolute risk if relevant | “As this was a cross-sectional study using logistic regression, associations were expressed as odds ratios with 95% confidence intervals. Absolute risk estimates were not calculated, as they are not directly estimable in this study design.” |
| Other analyses: Subgroups, interactions, sensitivity analyses (if applicable) | Not applicable |

# Discussion

| Checklist Item | Page Number(s) |
| --- | --- |
| Key results: Summarise key results with reference to objectives | 16 |
| Limitations: Discuss limitations, sources of bias/precision, generalisability | 19 |
| Interpretation: Provide cautious interpretation, considering objectives, limitations, evidence | 19-20 |
| Generalisability: Discuss external validity | 20 |

# Other Information

| Checklist Item | Page Number(s) |
| --- | --- |
| Funding: State source of funding and role of funders | 22 |
