# Supplementary material for: Associations between attitudes accepting of wife abuse and emotional abuse, forced heavy work, and food deprivation during pregnancy in Nepal: a cross-sectional study
Source: Glob Health Action. 2026 Jan 2;19(1):2603864. doi: 10.1080/16549716.2025.2603864 (PMC12777793; doi:10.1080/16549716.2025.2603864)
Supplement: Supplementary_file_containing_all_the_supplememtary_figures_and_tables.docx [file ZGHA_A_2603864_SM5398.docx]

Supplementary Table 1: Loadings of items on acceptance of wife abuse (exploratory factor analysis) and corrected item-total correlations (N = 2909).

| **Items** | **Mean** | **SD** | **Extraction values** | **Factor 1** | **Factor 2** | **Factor 3** | **Corrected item-total correlation** |
| --- | --- | --- | --- | --- | --- | --- | --- |
| She fails to prepare meals in time | .06 | .24 | .48 | .56 |  |  | .54 |
| She burns the meals | .04 | .19 | .54 | .54 |  |  | .59 |
| She fails to prepare tasty meals | .06 | .23 | .63 | .70 |  |  | .69 |
| She chats with a man | .13 | .33 | .48 | .64 |  |  | .51 |
| She argues with her husband | .08 | .26 | .56 | .71 |  |  | .63 |
| She does not complete her household work to his satisfaction | .08 | .26 | .56 | .66 |  |  | .65 |
| She disobeys him | .11 | .31 | .48 | .59 |  |  | .57 |
| She refuses to have sex / perform any sexual acts with him | .06 | .23 | .51 | .62 |  |  | .64 |
| She asks him whether he has other girlfriends | .09 | .29 | .45 | .58 |  |  | .57 |
| He suspects that she is unfaithful | .29 | .45 | .73 |  |  | .76 | .49 |
| He finds out that she has been unfaithful | .48 | .50 | .55 |  |  | .76 | .54 |
| She brings less or no dowry | .03 | .18 | .53 |  | .76 |  | .61 |
| She gives birth to daughters only and no son | .04 | .18 | .59 |  | .82 |  | .64 |
| His family tells him to do it | .05 | .21 | .67 |  | .68 |  | .53 |
| She neglects the children | .38 | .48 | .57 |  |  | .74 | .48 |
| She goes out without telling him | .24 | .42 | .44 |  |  | .61 | .44 |

Note: Factors were extracted using exploratory factor analysis with varimax rotation. All factor loading are significant at the .001 level. Cronbach’s alpha: Factor 1=0.861, Factor 2=0.763, Factor 3=0.705.
