# Supplementary material for: Associations between attitudes accepting of wife abuse and emotional abuse, forced heavy work, and food deprivation during pregnancy in Nepal: a cross-sectional study
Source: Glob Health Action. 2026 Jan 2;19(1):2603864. doi: 10.1080/16549716.2025.2603864 (PMC12777793; doi:10.1080/16549716.2025.2603864)
Supplement: Tables_with_their_respective_legends_clean copy.docx [file ZGHA_A_2603864_SM5397.docx]

Table 1. Questionnaire measures of domestic violence used in the study.

| **Types of abuse** | **Questions inquired** | **Response** | **Perpetrators (multiple responses)** |
| --- | --- | --- | --- |
| **Emotional abuse** | “*Since you’ve been married or living with a partner, have you been emotionally abused by someone in the family?” (Examples: Emotional abuse includes such acts as saying or doing something to humiliate you intentionally in front of others, the use of abusive words directed at you, threatening to hurt or harm you and your child/children, insulting you, deliberately making you feel bad about yourself, prohibiting you from visiting your mother’s home, etc.)* | Yes/No | husband, ex-husband, partner, mother-in-law, father-in-law, and other family members |
| **Forced heavy work** | “*Since you’ve been pregnant, have you been forced by someone in the family to do heavy physical work despite feeling unwell or exhausted? (Examples: Forced heavy physical work includes activities such as being made to carry a 20 liter of water jar or load of hay, forced to sit for a long period of time while washing clothes, lift sacks of rice, prolonged walking, etc.).* | Yes/No | husband, ex-husband, partner, mother-in-law, father-in-law, and other family members |
| **Food deprivation** | *“Since you’ve been pregnant, has someone in the family given you insufficient or no food to eat despite the availability of food and money in the household?” (Examples: not providing nutritious food such as eggs, fish, meat, fruits or hiding those foods).* | Yes/No | husband, ex-husband, partner, mother-in-law, father-in-law, and other family members |

Table 2: Socio-demographic characteristics of the participants (N = 2909)

| **Socio-demographic characteristics** | | **Total**  **n (%)** |
| --- | --- | --- |
| Study site | Dhulikhel Hospital | 1421 (48.8) |
|  | Kathmandu Medical College | 1488 (51.2) |
| Age (years) | 18-24 | 719 (24.7) |
|  | 25-29 | 1181 (40.6) |
|  | 30-49 | 1009 (34.7) |
| Primigravid | Yes | 1328 (45.7) |
|  | No | 1581 (54.3) |
| Currently married | Yes | 2892 (99.4) |
|  | No | 17 (0.6) |
| Independent income | Yes | 1206 (41.5) |
|  | No | 1703 (58.5) |
| Education | Low education | 619 (21.3) |
|  | High education | 2290 (78.7) |
| Family type | Nuclear | 1648 (56.7) |
|  | Joint | 1261 (43.3) |
| Ethnic group | Adhibasi/Janajati | 1034 (35.5) |
|  | Brahmin/Chhetri | 1519 (52.2) |
|  | Dalit | 130 (4.5) |
|  | Madhesi/ Muslim | 102 (3.5) |
|  | Others | 124 (4.3) |
| Settlement | Rural | 813 (27.9) |
|  | Urban | 2096 (72.1) |

Table 3: Prevalence of individual and overlapping forms of abuse among pregnant women (N = 2909)

| **Types of abuse reported** | **Yes**  **n (%)** | **No**  **n (%)** |
| --- | --- | --- |
| Emotional abuse only | 116 (4.0) | 2793 (96.0) |
| Forced heavy work only | 24 (0.8) | 2885 (99.2) |
| Food deprivation only | 22 (0.8) | 2887 (99.2) |
| Emotional abuse + Forced heavy work | 18 (0.6) | 2891 (99.4) |
| Emotional abuse + Food deprivation | 5 (0.2) | 2904 (99.4) |
| Forced heavy work + Food deprivation | 0 (0) | 2909 (100) |
| Emotional abuse + Forced heavy work + Food deprivation | 8 (0.3) | 2901 (99.7) |
| Any of the above (≥ 1 type of abuse reported) | 193 (6.7) | 2716 (93.3) |

Note: “Any of the above” refers to women reporting at least one of the three forms of abuse (EAFF), either alone or in combination.

Table 4: Perpetrators of emotional abuse, forced heavy work and food deprivation reported by pregnant women

| **Type of abuse** | **Perpetrator** | **N (%)** |
| --- | --- | --- |
| Emotional abuse | Husband | 37 (25.2) |
|  | Ex-husband | 4 (2.7) |
|  | Partner | 1 (0.7) |
|  | Mother-in-law | 55 (37.4) |
|  | Father-in-law | 24 (16.3) |
|  | Other family members | 74 (50.3) |
| **Total** |  | **195 (100)** |
| Forced heavy work | Husband | 14 (28) |
|  | Ex-husband | 1 (2) |
|  | Partner | 1 (2) |
|  | Mother-in-law | 19 (38) |
|  | Father-in-law | 6 (12) |
|  | Other family members | 23 (46.0) |
| **Total** |  | **64 (100)** |
| Food deprivation | Husband | 7 (20) |
|  | Ex-husband | 0 |
|  | Partner | 1 (2.9) |
|  | Mother-in-law | 13 (37.1) |
|  | Father-in-law | 1 (2.9) |
|  | Other family members | 17 (48.5) |
| **Total** |  | **39 (100)** |

Note: Multiple responses permitted. The total number of perpetrators exceeds the number of women affected because some women reported emotional abuse, forced heavy work, or food deprivation by more than one family member.

Table 5: Prevalence of acceptance of wife abuse overall and by attitudinal factors: domestic shortcomings, marital and patriarchal expectations, and transgressing expected female family roles (N = 2909)

| **Factor / Item** | **Yes**  **n (%)** | **No**  **n (%)** |
| --- | --- | --- |
| **Factor 1: Domestic shortcomings** | | |
| She fails to prepare meals in time | 182 (6.3) | 2727 (93.7) |
| She burns the meals | 114 (3.9 | 2795 (96.1) |
| She fails to prepare tasty meals | 164 (5.6) | 2745 (94.4) |
| She chats with a man | 365 (12.5) | 2544 (87.5) |
| She argues with her husband | 220 (7.6) | 2689 (92.4) |
| She does not complete her household work to his satisfaction | 224 (7.7) | 2685 (92.3) |
| She disobeys him | 315 (10.8) | 2594 (89.2) |
| She refuses to have sex / perform any sexual acts with him | 166 (5.7) | 2743 (94.3) |
| She asks him whether he has other girlfriends | 272 (9.4) | 2637 (90.6) |
| **Factor 2: Marital and patriarchal expectations** | | |
| She brings less or no dowry | 99 (3.4) | 2810 (96.5) |
| She gives birth to daughters only and no son | 103 (3.5) | 2806 (96.5) |
| His family tells him to do it | 136 (4.7) | 2773 (95.3) |
| **Factor 3: Transgressing expected female family roles** | | |
| He suspects that she is unfaithful | 850 (29.2) | 2059 (70.8) |
| He finds out that she has been unfaithful | 1404 (48.3) | 1505 (51.7) |
| She neglects the children | 1110 (38.2) | 1799 (61.8) |
| She goes out without telling him | 685 (23.5) | 2224 (76.5) |

Table 6: Association between attitudes accepting of wife abuse (factor 1, factor 2, factor 3), sociodemographic variables, and emotional abuse or forced heavy work or food deprivation (N = 2909)

| **Independent variable** | **Sig.** | **COR (95% CI)** | **Sig.** | **AOR (95% CI)** |
| --- | --- | --- | --- | --- |
| Factor 1 (Domestic shortcomings) | **<.001** | **1.98 (1.46-2.67)** | **.002** | **1.75 (1.23-2.50)** |
| Factor 2 (Marital and patriarchal expectations) | .21 | 1.38 (.83-2.29) | .50 | .82 (.47-1.43) |
| Factor 3 (Transgressing expected female family roles) | .59 | 1.08 (.80-1.47) | .20 | .80 (.57-1.12) |

Note: The final model presents attitudinal predictors only and is adjusted for age, marital status, education, family type, and settlement
